# Metal Nanoparticles to Combat *Candida albicans* Infections: An Update

**DOI:** 10.3390/microorganisms11010138

**Published:** 2023-01-05

**Authors:** Paulo Henrique Fonseca do Carmo, Maíra Terra Garcia, Lívia Mara Alves Figueiredo-Godoi, Anna Carolina Pinheiro Lage, Newton Soares da Silva, Juliana Campos Junqueira

**Affiliations:** 1 Department of Biosciences and Oral Diagnosis, Institute of Science and Technology, São Paulo State University (Unesp), São José dos Campos 12245-000, SP, Brazil; 2 Instituto René Rachou, Fiocruz Minas Gerais, Belo Horizonte 30190-002, MG, Brazil; 3 Department of Environmental Engineering, Institute of Science and Technology, São Paulo State University (Unesp), São José dos Campos 12245-000, SP, Brazil

**Keywords:** antifungal agent, metal nanoparticles, silver, gold, iron, *Candida albicans*

## Abstract

Candidiasis is an opportunistic mycosis with high annual incidence worldwide. In these infections, *Candida albicans* is the chief pathogen owing to its multiple virulence factors. *C. albicans* infections are usually treated with azoles, polyenes and echinocandins. However, these antifungals may have limitations regarding toxicity, relapse of infections, high cost, and emergence of antifungal resistance. Thus, the development of nanocarrier systems, such as metal nanoparticles, has been widely investigated. Metal nanoparticles are particulate dispersions or solid particles 10–100 nm in size, with unique physical and chemical properties that make them useful in biomedical applications. In this review, we focus on the activity of silver, gold, and iron nanoparticles against *C. albicans*. We discuss the use of metal nanoparticles as delivery vehicles for antifungal drugs or natural compounds to increase their biocompatibility and effectiveness. Promisingly, most of these nanoparticles exhibit potential antifungal activity through multi-target mechanisms in *C. albicans* cells and biofilms, which can minimize the emergence of antifungal resistance. The cytotoxicity of metal nanoparticles is a concern, and adjustments in synthesis approaches or coating techniques have been addressed to overcome these limitations, with great emphasis on green synthesis.

## 1. Introduction

Candidiasis is an opportunistic mycosis caused by *Candida* spp. and affects the oral cavity, skin, reproductive tract, and gastrointestinal tract [1,2,3,4]. *Candida* infections can also occur systemically and lead to life-threatening candidaemia [5,6]. The annual incidence of *Candida* infections is approximately four million cases worldwide [7] with a mortality rate about 40% [8,9], resulting in a significant economic burden owing to high drug costs and long hospital stay [10].

**Figure 1:**
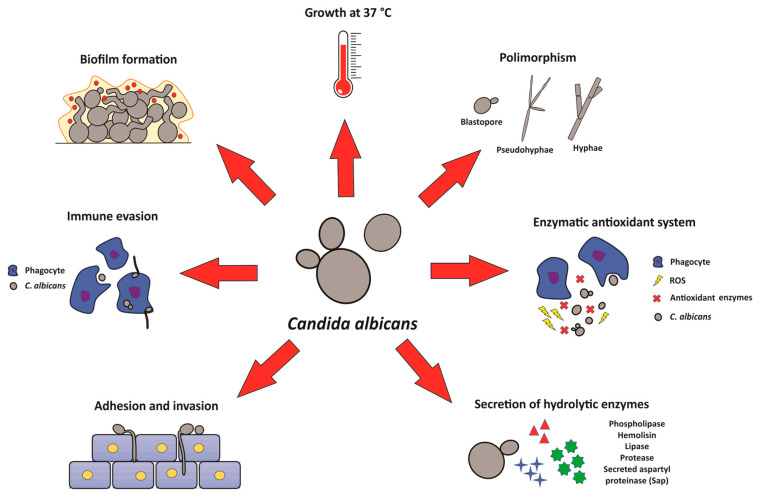
Major virulence factors of *C. albicans. C. albicans* is a fungal pathogen that is able to grow at 37 °C, transition from the yeast stage to hyphae or pseudohyphae (polymorphism), form biofilms on biotic and abiotic surfaces, evade the immune system by filamentation, adhere and invade tissues and surfaces, secrete several hydrolytic enzymes (phospholipase, haemolysin, lipase, protease, and mainly secreted aspartyl protease—Sap), and enzymes of the fungal antioxidant system (catalase, superoxide dismutase, glutathione peroxidase, and glutathione reductase). ROS: reactive oxygen species.

Among the aetiologic agents of candidiasis, *C. albicans* is the chief pathogen due to its high prevalence in infections and phenotypic plasticity [7,11]. The colonization and infection by *C. albicans* are influenced by fungal virulence factors that include the ability to grow at 37 °C, morphological transition, secretion of hydrolytic enzymes, haemolytic activity, tissue adhesion and invasion, evasion from the host immune system, filamentation ability, and biofilm formation [12,13] (Figure 1).

*C. albicans* forms biofilms under different environmental conditions, and on biotic and abiotic surfaces that include host tissues, medical devices, dentures, and catheters [14,15]. Biofilms are communities of yeast, pseudohyphae, and hyphae that are encapsulated in a self-secreted polymeric extracellular matrix [16]. *C. albicans* biofilms are complex structures that protect cells from the surrounding environment, providing a high level of resistance to conventional antifungal agents and host immune system components. These biofilms contribute to recurrent candidiasis and represents a challenge for antifungal therapy [17,18].

Usually, *C. albicans* infections are treated using antifungal drugs, including azoles, polyenes and echinocandins. However, these antifungals may be limited in terms of toxicity, limited spectrum, relapse of infections, route of administration, high cost, availability, and the emergence of antifungal resistant strains of *C. albicans* [19,20]. To overcome these limitations, several studies have sought to identify new therapeutic strategies for candidiasis with a focus on nanotechnology [21,22].

In this context, metal nanoparticles have been widely studied because of their intrinsic antifungal activity and potential to deliver antifungal drugs, with a focus on increasing the biocompatibility and effectiveness of these compounds [23,24,25]. In this review, we discuss different types of metal nanoparticles, mechanisms of action, methods of synthesis, cytotoxicity for host cells, and their use as drug delivery systems to treat candidiasis.

## 2. Metal Nanoparticles

The development of nanodrugs and nanocarriers has provided new perspectives in the biomedical field. Metal nanoparticles have received much attention because of their potential activity against fungal species of interest in the medical, pharmaceutical, and agricultural fields [26,27,28,29]. A wide range of nanoparticles with inorganic cores has been proposed, including noble metals, magnetic materials, and metal oxide nanoparticles [30].

Nanoparticles are particulate dispersions or solid particles 10–100 nm in size. They are defined as objects with three external nanoscale dimensions [31]. Metal nanoparticles have unique physical and chemical properties. Adjusting their synthesis process can modify their size, deformability, charge, hydrophobicity, and porosity, which can affect particle stability, biocompatibility, drug charge, circulation time, biodistribution, cellular localization, and internalization [23].

**Figure 2:**
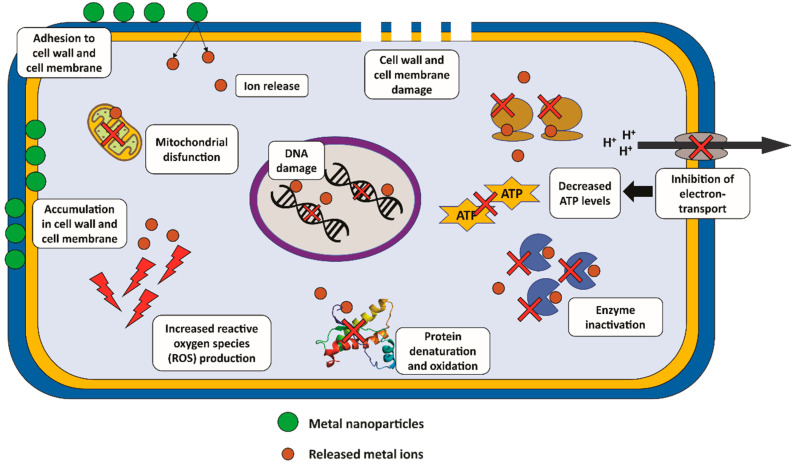
Main mechanisms of action of metallic nanoparticles against *C. albicans.* Metal nanoparticles exhibit activity against *C. albicans* by metal ion release, adhesion, accumulation and damage to the cell wall and cell membrane, inhibition of electron-transport with consequent reduction in ATP levels, enzyme inactivation, protein oxidation and denaturation, increased production of reactive oxygen species (ROS), mitochondria dysfunction, and DNA damage.

Metal nanoparticles exhibit antifungal activity through mechanisms involving the release of ions, oxidative and nitrosative stress, membrane and cell wall damage, enzymatic activity inhibition, gene expression regulation, decreasing ATP levels, DNA, protein, and mitochondrial dysfunction (Figure 2) [27,32,33,34].

Based on these properties, metal nanoparticles have broad applications in the drug delivery of antimicrobial agents [29,35,36,37]. The use of metal nanoparticles in combination with amphotericin B (AMB), caspofungin (CAS) and fluconazole (FCZ) may enhance its effectiveness against *C. albicans* [14,38,39]. Metal nanoparticles can play an important role in combating *C. albicans* strains resistant to antifungal drugs and biofilms formed on mucosa and medical devices [14,15]. Here, recent studies focused on the use of metal nanoparticles to combat *C. albicans* infections are highlighted, with a special focus on silver (Ag), gold (Au), and iron (Fe) nanoparticles.

### 2.1. Silver Nanoparticles

Silver nanoparticles (AgNPs) have been widely studied for their potential applications in biomedical areas such as anticancer drug nanocarriers, vaccines, and drug delivery systems [37,40]. Recent improvements in their properties of biocompatibility and stability through surface modifications make them candidates for carrying a large number of compounds with antifungal activity [35].

#### 2.1.1. AgNPs as Systems to Carrier Antifungal Drugs

AgNPs have been used as carrier systems for conventional antifungals such as voriconazole [41], nystatin [42], and AMB [43]. The use of AgNPs to carrier antifungals makes it possible to increase the efficacy of therapy with reductions in drug concentration, toxicity and cost [35].

Recently, Jia and Sun [44] evaluated FCZ alone and in combination with AgNPs on two FCZ-resistant *C. albicans* strains. AgNPs had a size ranging from 8 to 12 nm. The minimum inhibitory concentration (MIC) of FCZ decreased when it was used in combination with AgNPs against planktonic cells, indicating a synergistic effect between AgNPs and FCZ. Combination therapy also exhibited synergism against biofilms in the early stages of formation 6 and 12 h, but not in mature biofilms. In addition, exposure to AgNPs reduced ergosterol levels, impaired membrane structure by downregulating the *ERG1*, *ERG11*, and *ERG25* genes [45], and increasing ROS production, resulting in oxidative degradation and cellular damage [46,47]. Interestingly, these authors also verified that AgNPs treatment reduced the membrane content of Cdr1p and Cdr2p, and thus efflux pump activity. FCZ-resistant *C. albicans* strains usually exhibit overexpression or increased efflux pump activity, which reduces their susceptibility to antifungal agents [48,49]. In this case, exposure to AgNPs restored the FCZ susceptibility phenotype of the resistant strains. Finally, the combination treatment of AgNPs and FCZ improved the survival rate of infected mice, and markedly reduced the fungal burden in the liver and kidneys. Therefore, combination therapy was a useful strategy for the treatment of FCZ-resistant strains *in vitro* and *in vivo*.

Falcão et al. [14] developed poly(methacrylic acid)-silver nanoparticles (PMAA-AgNPs) capped with FCZ and evaluated their *in vitro* effects on *C. albicans* and mammalian cells. PMAA-AgNPs had a spherical shape, mean diameter of 15 nm, and a Zeta potential of −41.83 mV. Zeta potential is a significant physiochemical attribute of nanosystems that can predict their interactions with different surfaces, playing a crucial role in drug delivery systems [21]. PMAA-AgNPs and FCZ reportedly exhibited synergistic interactions against *C. albicans* strains resistant to FCZ, leading to a reduction in MIC values and inhibition of germ tube formation. PMAA-AgNPs were toxic in a dose-dependent manner to J774.A1 macrophages and fibroblasts, but the concentrations required to decrease cell viability were higher than those that inhibited *C. albicans* growth [14]. This combination therapy provided a convincing way to reduce the amount of antifungal drugs compared to antifungal monotherapy, and most importantly, overcome antifungal drug resistance in *C. albicans* [25,50].

While AgNPs exhibit promising results as drug delivery systems for antifungal agents, their potential cytotoxicity is a concern [51]. Therefore, development of AgNPs with antifungal activity should consider the toxicity levels and eco-friendly characteristics [52].

#### 2.1.2. Green Synthesis of AgNPs

Green synthesis is a promising approach in many aspects of modern nanobiotechnology. Green synthesis uses compounds of natural origin, such as proteins, alkaloids, flavonoids, and polyphenols, such as metal ion reducing and stabilizing agents to produce nanoparticles. Green synthesis is an eco-friendly and inexpensive method that can be easily incorporated into the industrial scale production of nanoparticles [53]. In addition to natural compounds, microbial cells and cell-free extracts of microorganisms can also be a source of reducing and stabilizing agents for green synthesis [52,54]. Metal nanoparticles synthetized using green methods that are coated with organic compounds can have increased biological activity and reduced toxicity [55].

Ahamad et al. [56] produced biogenic silver nanoparticles from *Anabaena variabilis* (AV-AgNPs), a prokaryotic photosynthetic filamentous cyanobacterium. The resulting AV-AgNPs were spherical and 11–15 nm in size. Antifungal activity against *C. albicans* planktonic cells and synergism with FCZ have been demonstrated. In a subsequent study, Ahamad and Bano [57] verified the capacity of AV-AgNPs to inhibit biofilms and suppress the filamentation of *C. albicans*. *C. albicans* cells exposed to AV-AgNPs were wrinkled and deformed, with nuclear abnormalities and membrane damage. These observations reinforce the ability of AV-AgNPs to damage *C. albicans* in both planktonic and biofilm stages.

Similarly, Abdallah and Ali [27] used the leaf extract of *Erodium glaucophyllum* to biosynthesize AgNPs (EG-AgNPs). The authors characterized the EG-AgNPs and assessed their antifungal effect against *C. albicans*. The EG-AgNPs exhibited a diameter of 50 nm, Zeta potential of –10 mV, and higher anti-*Candida* activity than AMB. The MIC value of EG-AgNPs and AMB was 50 and 100 μg/mL, respectively. In addition, EG-AgNPs completely eliminated the fungal burden within 10 h post-treatment, with complete elimination achieved within 20 h of AMB treatment. The dimorphic transition of *C. albicans* was inhibited by 56.36% at 25 μg/mL EG-AgNPs and the viability of biofilms was reduced by 52% at 50 μg/mL. EG-AgNPs applied at 50 μg/mL also decreased the enzymatic activity of both proteinases and phospholipases. Transmission electron microscopy (TEM) images revealed that EG-AgNPs treatment (50 μg/mL) led to modifications in the morphology of fungal cells, including cytoplasmic disintegration, vacuolation, perinuclear, and granular nuclear alteration. In addition, the *C. albicans* cell wall was swollen and the outer layer appeared to be separated from the cell [12,58]. EG-AgNPs cytotoxicity was not evident for the human gingival fibroblast (HGF-1) when used at concentrations up to 150 μg/mL. The efficiency of topical treatment with 50 μg/mL of EG-AgNPs was examined in an *in vivo* mouse model of oral candidiasis. Treatment with EG-AgNPs resulted in a reduction in the number of fungal cells and lesions in the tongue dorsum, suggesting the potential use of EG-AgNPs in the treatment of oral candidiasis.

Alqarni [59] used Gum acacia, which is a material enriched in polysaccharide from acacia plants. Gum acacia was used as a dispersal and reduction agent to synthetize rutin-AgNPs. Rutin, also known as vitamin P or rutoside, exhibits several pharmacological effects, including anti-*Candida* activity [60]. Rutin-AgNPs had an average size of 59.67 nm, and Zeta potential of –11.2 mV. Rutin-AgNPs were incorporated into base gels to produce rutin-loaded AgNPs gel to evaluate their activity in skin candidiasis. Both formulations exhibited antifungal activity against *C. albicans*. However, this activity was enhanced in the rutin-loaded AgNPs gel. This gel increased drug release, probably due to the capsizing effects of rutin on the formed AgNPs. Altogether, the effectiveness of this formulation offers new perspectives for the topical delivery of antifungal agents for the treatment of *Candida* wound infections.

Miškovská et al. [61] synthesized AgNPs using *Vitis vinifera* cane extract (VV-AgNPs). These nanoparticles had various shapes, with an average size ranging from 34.43 to 101.63 nm, and a Zeta potential ranging from –30.04 to –21.24 mV. VV-AgNPs were active against planktonic cells and biofilms of *C. albicans* at a concentration of 20 µg/mL. Scanning electron microscopy (SEM) images demonstrated that *C. albicans* cells treated with VV-AgNPs (5 µg/mL) visibly adhered to the surface, while other cells were clearly disrupted, reinforcing the antifungal effect of VV-AgNPs against *C. albicans*.

### 2.2. Gold Nanoparticles

Gold nanoparticles (AuNPs) have also been extensively studied because of their good physicochemical properties, chemical resistivity, ease of synthesis, and minimal size [62,63,64]. The Food and Drug Administration (FDA) approval for the use of AuNPs in various biomedical applications has boosted research and development focused on these nanoparticles [65]. The unique properties of AuNPs include adjustable size, shape, surface properties, biocompatibility, low cytotoxicity, and high stability [24]. Furthermore, they can be easily combined or recovered with several biomolecules, including peptides, enzymes, and DNA, enabling their use in inducing drug stability and reducing adverse effects [66,67]. While AuNPs exhibit a variety of biomedical effects, cytotoxicity remains a concern [68]. Strategies that include changing the charge, size, and dispersion of AuNPs can be easily employed to minimize toxicity, and/or enhance antifungal activity [65,69].

#### 2.2.1. AuNPs as Carrier Systems for Antifungal Drugs

AuNPs have been typically investigated for the delivery of conventional antifungals. For example, Salehi et al. [38] formulated the antifungal caspofungin by incorporating it into AuNPs (CAS-AuNPs). CAS, a member of the echinocandin class, inhibits the biosynthesis of the fungal cell wall component β-1,3-D-glucan [70,71,72]. In the study, resulting CAS-AuNPs had a spherical shape with an average size of 20 nm and the Zeta potential of –38.2 mV. CAS-AuNPs exhibited anti-*Candida* activity at lower concentrations than CAS alone. SEM images demonstrated membrane and cell wall damage in yeast cells after exposure to CAS-AuNPs, indicating the improved antifungal activity associated with reduced antifungal concentrations and decreased toxicity [38].

Similarly, Hamad et al. [73] described the formation of functionalized AuNPs with poly(ethylene) glycol (PEG) and FCZ. The resulting PEG-FCZ-AuNPs were loaded into a poloxamer hydrogel (PEG-FCZ-AuNP hydrogel). PEG-FCZ-AuNP formed nanorods, with a Zeta potential of +1.6 mV, and an average length of 80 nm. Hydrogel loading did not affect the size and Zeta potential of the PEG-FCZ-AuNP. The MIC value of PEG-FCZ-AuNPs was 0.12 nM, which corresponded to a nine-fold reduction compared to FCZ alone. TEM images revealed that the nanoformulation enhanced the delivery of FCZ to the cell wall and accelerated its cellular uptake. The exposure of *C. albicans* to the PEG-FCZ-AuNP hydrogel resulted in a 1.3 log reduction in the viability of fungal cells. The cytotoxicity of PEG-FCZ-AuNP was evaluated against CCD-1064Sk human dermal fibroblasts. Exposure of these cells to PEG-FCZ-AuNPs for 24 h maintained cell viability by more than 75% at the MIC value against *C. albicans*, suggesting that it is a promising approach for the effective treatment of mucosal candidiasis.

#### 2.2.2. Chitosan-AuNPs as Anti-*C. albicans* Nanosystems

The association of AuNPs with organic polymers such as chitosan has also been studied. Chitosan is a natural cationic polysaccharide used as a biopolymer for the synthesis of colloidal nanoparticles. Chitosan act as a reducing and stabilizing agent [74], and exhibits intrinsic *in vitro* anti-*Candida* activity owing to its polycationic nature. Interaction with the negatively charged cell membrane of microorganisms via electrostatic interactions leads to extensive cell surface modifications [75,76]. Furthermore, chitosan exhibits anti-inflammatory activity, good biocompatibility, mucoadhesive and haemostatic properties, and the capacity to modulate fibroblast growth factors. These attributes have led to the use of chitosan as a wound dressing material [77].

Hashem et al. [69] synthetized spherical chitosan-based AuNPs (Chi-AuNPs) with an average particle size ranging from 20 to 120 nm and a Zeta potential of −52.39 mV. Evaluation of the antifungal effect of Chi-AuNPs was assessed for *C. albicans* using two methods. In the first method, Chi-AuNPs produced a 25 mm inhibition zone when applied at 2000 µg/mL, with a MIC value of 62.5 µg/mL. In the microdilution toxicity assays, Chi-AuNPs were non-toxic to the BJ-1 normal human skin cell line, with a cytotoxic concentration for 50% of the cells of 111.10 µg/mL. The findings highlight the feasibility of Chi-AuNPs as a promising agent against *C. albicans* infections [69].

Yadav et al. [21] designed tyrosol-functionalized chitosan gold nanoparticles (Chi-TY-AuNPs). The intent was to use the Chi-TY-AuNPs as an alternative treatment strategy for *Candida* infections. Tyrosol is a molecule produced by *C. albicans*; it is involved in quorum sensing, which stimulates hyphal formation. In contrast, exogenous tyrosol administration can inhibit planktonic cells and biofilms of *C. albicans* and enhance their susceptibility to antifungal agents [78]. However, the exogenous mechanism underlying the antagonistic action of tyrosol against *C. albicans* has not been fully elucidated. Yadav et al. [21] reported that Chi-TY-AuNPs had a Zeta potential of +45.5 mV and were spherical, with an average diameter in the range of 10−15 nm. These nanoparticles showed activity against *C. albicans* at 200 μg/mL, and the minimum fungicidal concentration was 800 μg/mL. Chi-TY-AuNPs were also able to inhibit biofilm development and eradicate mature biofilms of *C. albicans* at 200 and 400 μg/mL, respectively. In addition, Chi-TY-AuNPs inhibited germ tube development and transition from yeast to hyphae at a subinhibitory concentration of 200 μg/mL. The ability of Chi-TY-AuNPs to inhibit *C. albicans* was associated with increased ROS production. Yadav et al. [21] also evaluated the cytotoxicity of Chi-TY-AuNPs to NIH-3T3 fibroblast cells, and reported the absence of reduced cell viability, even when these nanoparticles were tested in higher concentrations. The finding highlighted the good biocompatibility of Chi-TY-AuNPs.

#### 2.2.3. Green Synthesis of AuNPs

As with AgNPs, green methods have been used to synthetize AuNPs. Kareem, Samaka and Abdulridha [28] produced gold nanoparticles using olive leaf extract (OL-AuNPs). The OL-AuNPs displayed a particle size of 29.16 nm. These nanoparticles inhibited *C. albicans* growth at 40.77 ng/mL. The antifungal effects on the cutaneous candidiasis infections were evaluated. OL-AuNPs showed good pharmacological efficacy in the treatment of murine cutaneous candidiasis, with superior efficacy compared to the antifungal nystatin. The candidiasis lesions of the group treated with 326.12 ng/mL OL-AuNPs were entirely cured three days post-treatment, confirming the anti-*Candida* effects of OL-AuNPs *in vitro* and *in vivo*.

In a thorough study, Cruz et al. [18] evaluated the ability of fifteen ethnobotanical crude extracts from the leaves, roots and/or bark of *Hydrocotyle vulgaris*, *Eluesine indica*, *Mikania micrantha*, *Dillenia philippinensis*, *Ceiba pentandra*, *Cymbopogon winterianus*, *Senna alata*, *Urena lobata*, *Premna odorata*, *Stachytarpeta jamaicensis*, *Diplazium esculentum*, and *Phyllanthus urinaria* as well as their gold nanoparticles (CE-AuNPs) on biofilms and quorum sensing-related gene expression. The CE-AuNPs exhibited a variety of shapes and sizes depending on the crude extract used. None of the extracts or their CE-AuNPs exhibited antifungal activity against planktonic *C. albicans*. However, 14 CE-AuNPs decreased *C. albicans* biofilm formation at concentrations of 67.00 to 80.00 µg/mL. The expressions of *BCR1* and *HSP90* were downregulated after exposure to 13 crude extracts, and to 14 CE-AuNPs. *BCR1* and *HSP90* genes act as transcription factors involved in biofilm formation. *BCR1* downregulation affected the development of biofilms as well as the production of polymeric extracellular matrix [79,80]. Apparently, the extracts and CE-AuNPs may acted as quorum sensing inhibitor molecules that blocked the *BCR1* and *HSP90* pathways [81,82]. It is possible that the difference in CE-AuNPs activities was influenced by the composition of the extracts, and by the final nanoparticle size.

While not as well studied as AgNPs, available data indicate that AuNPs have effective antifungal activity, and so may be a key strategy for drug development against *C. albicans* infections.

### 2.3. Iron Nanoparticles

Iron oxide nanoparticles (IONP) are recognized by their high chemical and thermal stabilities, low cost, very high area-to-volume and area-to-weight ratios, and superparamagnetic behaviour. These attributes are valuable for biomedical applications [83,84]. Accordingly, IONPs have been explored as drug delivery systems to increase drug effectiveness and reduce therapeutic drug concentrations [36]. Ferumoxytol is an intravenous IONP formulation that is intravenously administrated to treat anaemia in patients with chronic kidney disease. Ferumoxytol is approved by the FDA, Health Canada, and other agencies to treat anaemia [85,86], and has also been proposed as a candidate for drug repurposing therapy owing to its excellent inherent properties and good safety and clearance profiles [85]. For example, topical ferumoxytol was able to disrupt oral biofilms by generating ROS and killing bacteria by membrane disruption, demonstrating the potential to prevent dental caries [87].

The antimicrobial activity of IONPs has also been proven against fungal species, such as *C. albicans* [33,88,89]. While there are concerns related to biocompatibility and toxicity, these characteristics can be modified by altering their size, shape, stability, and coating [90].

IONPs can be functionalized with different organic and inorganic compounds to modify their general properties, particularly their antimicrobial properties [91]. For example, Arias et al. [92] synthetized iron nanoparticles functionalized with chitosan as nanocarriers for the antifungal miconazole (IONP-Chi-MCZ). The resulting IONP-Chi-MCZ had a diameter < 50 nm and promoted an 8-fold reduction in *C. albicans* planktonic cells compared to miconazole. In contrast, IONP and quitosan alone did not inhibit *C. albicans* growth at 140 μg/mL, and the combination of IONP, quitosan, and miconazole was characterized as synergistic against *C. albicans*. IONP-Chi-MCZ also reduced the metabolic activity and viability of *C. albicans* biofilms. However, treatment with IONP-Chi-MCZ did not affect the protein, carbohydrate, or DNA content of the polymeric extracellular matrix.

Similarly, Balabathula et al. [29] designed IONP functionalized with bovine serum albumin and targeted amphotericin B (AMB-IONP). The synthetized AMB-IONP were spherical, monodisperse, had ≤36 nm in size, and a Zeta potential of −20 mV. AMB-IONPs exhibited improved efficacy (16–25-fold) compared to AMB against *C. albicans*. In addition, TEM and confocal laser scanning microscopy revealed intracellular trafficking of AMB-IONPs inside fungal cells, confirming successful drug delivery. TEM also revealed that the AMB-IONPs were localized inside the cytoplasm or near the cell wall and cell membrane, indicating that the uptake pathway mechanism of AMB-IONPs in *C. albicans* was controlled by receptor-mediated endocytosis. AMB-IONP treatment also led to the disorganization of the cytoplasm with deformed nuclei and other intracellular components, along with a significant increase in the number of vacuoles [29].

### 2.4. Other Metal Nanoparticles

In addition to the traditional silver, gold, and iron nanoparticles, several other metal nanoparticles have been investigated for the *in vitro* and *in vivo* treatment of *C. albicans* infections. Nanomaterials such as zinc oxide (ZnO), titanium dioxide (TiO_2_), zirconium dioxide (ZrO_2_), and copper (cupric oxide [CuO] and cuprous oxide [Cu_2_O]) have been used successfully to inhibit and kill *C. albicans* cells [93,94,95].

Padmavathi et al. [13] synthetized oxide copper nanoparticles (CuO-NP and Cu_2_O-NP) having an average size of 10.7 nm and 36 nm, respectively, and a Zeta potential of −38.35 mV, and +7.9 mV, respectively. CuO-NPs and Cu_2_O-NPs completely inhibited *C. albicans* growth at 150 and 200 µg/mL, respectively. Furthermore, CuO-NPs and Cu_2_O-NPs showed significant anti-biofilm activity against *C. albicans* at 1 µg/mL, which was comparable to that of AMB [96]. Macromorphologically, Cu_2_O-NP treatment decreased spore and hyphal formation on solid agar media. Microstructural analysis demonstrated that exposure of *C. albicans* to Cu_2_O-NPs led to cell shrinkage, possibly due to the treatment-induced increase in ROS production. This enhanced activity was attributed to the positive surface charge of Cu_2_O-NPs, since *C. albicans* cells are electronegative; their cell walls effectively bind positively charged ions, facilitating electrostatic interactions [97,98]. The CuO-NPs were shown to be internalized more than were Cu_2_O-NPs, probably because of their smaller size. Both CuO-NPs and Cu_2_O-NPs reduced ergosterol production in *C. albicans*, and downregulated the genes involved in morphogenesis and virulence.

Aati, Shrestha and Fawzy [99] synthesized zirconium dioxide nanoparticles (ZrO_2_NPs) and studied their antimicrobial and cytotoxic effects in a modified three-dimensional printed resin, which is routinely used in dentistry. The diameters of ZrO_2_NPs ranged from 20 to 40 nm before and after functionalization. The metabolic activity of *C. albicans* was remarkably reduced by the addition of ZrO_2_NPs, even after aging for three months. SEM confirmed the reduction in *C. albicans* biofilm viability when the resin was modified with ZrO_2_NPs. This antifungal activity may be related to the ability of ZrO_2_NPs to produce ROS and interfere with cell functions, leading to deformation of the fungal hyphae [100,101]. The authors measured cytotoxicity to human oral fibroblast. The higher concentration of ZrO_2_NPs used slightly reduced the number of viable cells, whereas low concentrations did not affect cell viability. Importantly, cell viability was re-established after aging for 3 months. Resin modified with ZrO_2_NPs exhibited a critical ability to inhibit biofilm formation without inducing any adverse cellular effects.

Seeking to refine the biomaterials used in dentistry, AlQahtani et al. [102] designed titanium dioxide nanoparticles and added them to polymethylmethacrylate (PMMA-TiO_2_NPs). Polymethylmethacrylate (PMMA) is commonly used as a denture base material owing to its low cost, reduced weight, colour-matching ability, and ease of finishing and polishing [95,103]. TiO_2_NPs exhibited a spherical shape with an average diameter of 26 nm. PMMA-TiO_2_NPs reduced *C. albicans* adhesion, probably because of the antifungal properties of TiO_2_NPs [104,105].

Taken together, these studies indicate that the successful use of different types of metallic nanoparticles in biomedical applications demands modulation of their properties to maximize the antifungal effect and decrease its toxicity [106].

### 2.5. Bimetallic Nanoparticles

Based on the antifungal activity exhibited by AgNPs, AuNPs, INOPs and other metal nanoparticles, several studies have addressed the use of bimetallic nanoparticles. In addition to antifungal drugs or natural biomolecules, metallic nanoparticles may be functionalized with different inorganic compounds, resulting in core–shell nanoparticles and bimetallic nanoparticles. In this context, a variety of metals, such as zinc (Zn), nickel (Ni), manganese (Mn), dysprosium (Dy), and copper (Cu) have been associated with AgNPs and IONPs to investigate their effects on *C. albicans*.

Padilla-Cruz et al. [25] synthesized AgNPs, iron (Fe), and Ag–Fe bimetallic nanoparticles (Ag-FeNPs) using *Gardenia jasminoides* extract as the reducing agent. The synthesized nanoparticles displayed homogenously distributed spherical core–shell structures with an average diameter of 13 nm and were magnetic. The activities of AgNPs, FeNPs, and Ag-FeNPs were evaluated. The MIC of AgNPs was 125 ppm and FeNPs exhibited no activity against *C. albicans* at the maximum concentration tested (250 ppm). Interestingly, Ag-FeNPs showed fungicidal activity against *C. albicans* at 62.5 ppm and were biocompatible. The findings indicate the potential of Ag-FeNPs as a nanomaterial for biomedical applications.

Other studies have evaluated the antifungal and cytotoxic activities of different silver chromite (Ag-CrNPs) nanocomposites [107]. Nanocomposites are hybrid materials in which at least one component has nanometric dimensions [108]. The synthetized Ag-CrNPs were spherical, with an average particle size of 93.14 nm. In addition, they showed significant antifungal activity against *C. albicans*, and concentrations < 31.25 μg/mL were not cytotoxicity against Vero Cells.

Kamli et al. [22] synthetized silver and nickel bimetallic nanoparticles (Ag-NiNPs) using an extract of *Salvia officinalis* leaves. Ag-NiNPs were generated as semi-spherical agglomerated clusters that harboured smaller and larger nanoparticles ranging in average size from 31.84 to 47.85 nm. Importantly, Ag-NiNPs showed potent antifungal activity against *C. albicans* strains resistant and susceptible to FCZ with effective concentrations ranging from 0.19 to 1.56 µg/mL. These nanoparticles also decreased hyphae production and biofilm formation. In addition to antifungal effects at low concentrations, Ag-NiNPs acted synergistically with FCZ against *C. albicans,* re-establishing the susceptibility of the fungus to FCZ. Furthermore, Ag-NiNPs modulated multi-drug resistance efflux transporters reducing ATP-dependent efflux pumps (ABC superfamily) in *C. albicans* at 1.56 µg/mL. Exposure to Ag-NiNPs also resulted in disruption of the *Candida* cell membrane accompanied by the generation of ROS, as confirmed by SEM. Cells treated with Ag-NiNPs exhibited a variety of sizes and shapes, surface depression, and leakage of intracellular material, reflecting the antifungal effect of Ag-NiNPs.

Based on the promising optoelectronic, electrochemical, catalytic and antibacterial properties of tin (Sn) and tin dioxide (SnO_2_) nanomaterials [109,110], and the well-established antimicrobial activity of AgNPs, Pandey et al. [111] evaluated the antifungal activity of bimetallic (Ag/Sn-SnO_2_) composite nanoparticles (Ag/Sn-SnO_2_NPs). Ag/Sn-SnO_2_NPs formed with a rod or needle shape, with ranging in size from 1 to 18 nm. The Zeta potential varied from −18.4 to −17.3 mV. Ag/Sn-SnO_2_NPs prepared easily and inexpensively inhibited *C. albicans. In silico* molecular docking evaluations of adhesion and invasion revealed that Ag/Sn-SnO_2_NPs exhibited interaction with several target amino acid residues of lanosterol 14-α-demethylase, an enzyme involved in the biosynthesis of ergosterol [112]. This binding directly inhibited the growth of *C. albicans* strains. Ag/Sn-SnO_2_NPs also interacted with Als3, Hsp90, and Cyp51, which are related to the growth of *C. albicans,* biofilm formation and tissue invasion [113], suggesting that the Ag/Sn–SnO_2_NPs exhibited potential inhibitory properties by targeting several proteins in different molecular pathways.

Sayed, Abdelsalam and El-Bassuony [114] developed several types of iron bimetallic nanoparticles to obtain spinel nanoferrites with diameters ranging from 22 to 97 nm. Among them, nickel-zinc-iron nanoparticles (Ni-Zn-IONPs) and manganese-zinc-dysprosium-iron nanoparticles (Mn-Zn-Dy-IONPs) exhibited activity against *C. albicans* at concentrations of 200 [34], and 8 mg/mL [115], respectively. Ni-Zn-IONPs had nanomagnetic surfaces containing nanospheres and nanosheets with an average particle size of 30 nm. Exposure of *C. albicans* to Ni-Zn-IONPs prevented fungal growth and increased leakage of intracellular proteins. Furthermore, Ni-Zn-IONPs increased ROS production, resulting in DNA and mitochondrial damage [34]. Mn-Zn-Dy-IONPs adopted a cubic shape with an average particle size < 20 nm. SEM demonstrated that exposure of *C. albicans* to Mn-Zn-Dy-IONPs resulted in deformed and distorted cells, indicating the loss of cell membrane integrity, which resulted in cell death [116]. Al-Jameel et al. [115] hypothesized that Mn-Zn-Dy-IONPs could bind to the cell surface and target ergosterol.

Ansari et al. [117] synthetized cube-shaped nickel-copper-zinc-iron nanoparticles (Ni-Cu-Zn-IONP) with an average size between 10 and 19 nm. Ni-Cu-Zn-IONP significantly reduced the number of planktonic cells of *C. albicans* at 2 mg/mL and inhibited biofilm formation at 1 mg/mL. Interestingly, SEM demonstrated inhibition of hyphae in treated biofilms with severely damaged treated yeast cells, including deformation, distortion and separation of the cell wall and membrane, indicative of a significant loss of membrane integrity. TEM revealed complete lysis of yeast cell and confirmed in SEM data. The inhibition of *C. albicans* was probably due to the adsorption and penetration of Ni-Cu-Zn-IONPs by yeast cells, which may have led to the disorganization of the cell membrane and wall, followed by the leakage of various cytoplasmic contents [116,118,119].

These collective findings confirmed that bimetallic nanoparticles can have antifungal activity against *C. albicans* strains via multiple mechanisms. These antifungal effects can be attributed to the unique physicochemical properties of each type of metal nanoparticles that, when combined, can interact with fungal cells through different pathways [120].

### 2.6. Emerging Concerns and Future Directions for Metal Nanoparticles

The antifungal activity against *C. albicans* and physical-chemical characteristics of metallic nanoparticles discussed in this review are summarized in the Table 1. The physical–chemical properties of nanoparticles have been considered key factors for their biological applications [121]. It is known that variations in physical and chemical parameters have a significant effect on cellular uptake and antimicrobial efficacy [121,122]. Metal nanoparticles are actively incorporated into the microbial cell through different endocytic pathways, and their size, shape and surface chemistry can influence the internalization process [121,123]. Typically, smaller nanoparticles have higher antimicrobial activity, while larger NPs require specific functionalization to increase their delivery into cells [62,124]. While there is no consensus about the optimum size that maximizes the cellular uptake, it has been suggested that small sized silver (5, 9, 10, 12, and 13.5 nm), gold (8.4 nm), zinc-oxide (12 nm), and titanium-oxide (12 and 17 nm) nanoparticles have high antimicrobial activities against bacterial cells [125]. However, little is known about the optimum size and other physical-chemical properties of metal nanoparticles that can maximize the cellular uptake by *Candida albicans,* and additional studies are required in this field.

Another issue that requires more investigation is the action of metal nanoparticles on polymicrobial biofilms. In these biofilms, *C. albicans* and bacterial species can establish synergistic interactions that provide protection to one or both species against antimicrobial agents and make polymicrobial biofilms more resistant than those formed by a single species. Competitive interactions can also be established between fungus and bacteria through the production of quorum sensing molecules [126]. Some microorganisms can secrete small molecules specialized in metal uptake named metallophores, which represent an important mechanism in the competitive interactions since iron, zinc and other metal ions are essential for the microbial survival. The most well-characterized metallophore family is the siderophore with high affinity for iron. Siderophores molecules are synthesized within the microbial cytoplasm and exported to the extracellular environment to scavenge the iron, then the siderophore-iron complex is transported into the periplasm by specific transporters [127,128]. Interestingly, it was reported that iron-based nanoparticles (Fe_3_O_4_ NPs) with antimicrobial activity were able to penetrate in Gram-negative bacteria through the siderophore transporters on the outer membrane [129]. While it is unclear whether *C. albicans* synthetizes its own siderophores, it possesses siderophore transporters and can take iron by siderophores produced by other microorganisms (xenosiderophores) [130,131]. These data instigate future studies to understand the role of metallophores in the antifungal activity of metal nanoparticles on *Candida* cells and polymicrobial biofilms.

Attention must also be given to possible adverse effects of metal nanoparticles. While most studies indicate that the toxicity of metal nanoparticles for mammalian cells is low, translation to clinical applications will require additional data concerning their possible accumulation in body tissues and adverse effects [132]. The absorption of metal ions by the mucosal surface can be associated with localized and generalized argyria, ocular irritation, contact dermatitis, and genotoxic, hepatic, renal, neurological, and haematological effects [133]. Thus, regulatory clarity concerning the use of metal nanoparticles is urgently needed. It is essential to encourage the development of clinical trials that use metal nanoparticles with proven in vitro and in vivo efficacy. These studies are crucial for the evaluation, knowledge, and follow-up of their effects on the body and environment.

## 3. Conclusions

Several *in vitro* and *in vivo* studies have shown the efficacy of metal nanoparticles in combating *C. albicans* at both planktonic and biofilm stages. Promisingly, most of the synthesized silver, gold, iron, and other metal nanoparticles inhibit the growth and lessen virulence of *C. albicans*, improving the development of antifungal resistance. Some studies have also demonstrated the *in vivo* efficacy of metal nanoparticles on oral and cutaneous candidiasis in rodent models, reinforcing their potential use as a candidate for the topical treatment of superficial candidiasis.

For clinical applications, improvements and adjustments in the antifungal activity and cytotoxicity properties of metal nanoparticles are still needed. To overcome these limitations, most studies have focused on modifications of the synthesis parameters and development of coating methods. Green synthesis and bimetallic coating are being intensively studied. The development of metal nanosystems could be a key strategy to circumvent problems related to antifungal resistance and recurrent candidiasis.

## Figures and Tables

**Table 1:** Characteristics and main activities of metal nanoparticles against *C. albicans* included in the study.

Nanoparticle	Antifungal Compounds	Size (nm)	Zeta Potential (mV)	Main Activities Against *C. albicans*	Reference
Silver	FCZ	8 to 12	ND	- Growth inhibition, anti-biofilm *in vitro* - Downregulation of ergosterol biosynthesis genes - ROS production - Reduction in efflux pump activity - Antifungal activity *in vivo*	[44]
	PMAA	15	−41.83	- Growth inhibition *in vitro* - Germ tube inhibition	[14]
	*Anabaena variabilis*	11 to 15	ND	Growth inhibition *in vitro*	[56]
	*A. variabilis*	11 to 15	ND	Anti-biofilm *in vitro*	[57]
	*Erodium glaucophyllum*	50	–10	- Growth inhibition, anti-biofilm *in vitro* - Enzymatic activity reduction - Filamentation inhibition - Antifungal activity *in vivo*	[27]
	Rutin	59.67	–11.2	Growth inhibition *in vitro*	[59]
	*Vitis vinifera*	34.43 to 101.63	–30.04 to –21.24	Growth inhibition, anti-biofilm *in vitro*	[61]
Gold	CAS	20	–38.2	Growth inhibition *in vitro*	[38]
	PEG and FCZ	80	+1.6	Growth inhibition *in vitro*	[73]
	Chitosan	20 to 120	−52.39	Growth inhibition *in vitro*	[69]
	Chitosan and tyrosol	10 to 15	+45.5	- Growth inhibition, anti-biofilm *in vitro* - Germ tube and morphogenesis inhibition - ROS production	[21]
	Olive leaf extract	29.16	ND	- Growth inhibition *in vitro* - Antifungal activity *in vivo*	[28]
	Crude extracts	ND	ND	- Anti-biofilm *in vitro* - Downregulation of biofilm formation genes	[18]
Iron	Chitosan and miconazole	<50	ND	- Growth inhibition *in vitro* - Metabolic activity reduced	[92]
	Bovine serum albumin and AMB	≤36	−20	- Growth inhibition *in vitro*	[29]
Other	Copper oxide	10.7 to 36	−38.35 to +7.9	- Growth inhibition and anti-biofilm *in vitro* - Morphogenesis inhibition - ROS production - Downregulation of morphogenesis and virulence genes	[13]
	Zirconium dioxide	20 to 40	ND	- Reduction of metabolic activity - Anti-biofilm *in vitro* - ROS production	[99]
	PMMA and titanium dioxide	26	ND	Reduction of adhesion	[102]
Bimetallic	Silver and iron	13	ND	- Growth inhibition *in vitro*	[25]
	Silver and chromium	93.14	ND	- Growth inhibition *in vitro*	[107]
	Silver, nickel and *Salvia officinalis*	31.84 to 47.85	ND	- Growth inhibition and anti-biofilm *in vitro* - Morphogenesis inhibition - Downregulation of efflux pump genes - ROS production	[22]
	Tin dioxide	1 to 18	−18.4 to −17.3	Growth inhibition and anti-biofilm *in vitro*	[111]
	Nickel, zinc, manganese, dysprosium and iron	20 to 30	ND	- Growth inhibition *in vitro* - ROS production	[114]
	Nickel, copper, zinc and iron	10 to 19	ND	Growth inhibition and anti-biofilm *in vitro*	[117]

ND: not determined.

## Data Availability

Not applicable.
